# Involvement of the Post-Transcriptional Regulator Hfq in *Yersinia pestis* Virulence

**DOI:** 10.1371/journal.pone.0006213

**Published:** 2009-07-10

**Authors:** Jing Geng, Yajun Song, Lei Yang, Yanyan Feng, Yefeng Qiu, Gang Li, Jingyu Guo, Yujing Bi, Yi Qu, Wang Wang, Xiaoyi Wang, Zhaobiao Guo, Ruifu Yang, Yanping Han

**Affiliations:** 1 State Key Laboratory of Pathogen and Biosecurity, Beijing Institute of Microbiology and Epidemiology, Beijing, China; 2 Laboratory Animal Center, Academy of Military Medical Sciences, Beijing, China; Theodor-Boveri-Institut fur Biowissenschaften, Wurzburg, Germany

## Abstract

**Background:**

*Yersinia pestis* is the causative agent of plague, which is transmitted primarily between fleas and mammals and is spread to humans through the bite of an infected flea or contact with afflicted animals. Hfq is proposed to be a global post-transcriptional regulator that acts by mediating interactions between many regulatory small RNAs (sRNAs) and their mRNA targets. Sequence comparisons revealed that *Y. pestis* appears to produce a functional homologue of *E. coli* Hfq.

**Methodology and Principal Findings:**

Phenotype comparisons using *in vitro* assays demonstrated that *Y. pestis* Hfq was involved in resistance to H_2_O_2_, heat and polymyxin B and contributed to growth under nutrient-limiting conditions. The role of Hfq in *Y. pestis* virulence was also assessed using macrophage and mouse infection models, and the gene expression affected by Hfq was determined using microarray-based transcriptome and real time PCR analysis. The macrophage infection assay showed that the *Y. pestis hfq* deletion strain did not have any significant difference in its ability to associate with J774A.1 macrophage cells. However, *hfq* deletion appeared to significantly impair the ability of *Y. pestis* to resist phagocytosis and survive within macrophages at the initial stage of infection. Furthermore, the *hfq* deletion strain was highly attenuated in mice after subcutaneous or intravenous injection. Transcriptome analysis supported the results concerning the attenuated phenotype of the *hfq* mutant and showed that the deletion of the *hfq* gene resulted in significant alterations in mRNA abundance of 243 genes in more than 13 functional classes, about 23% of which are known or hypothesized to be involved in stress resistance and virulence.

**Conclusions and Significance:**

Our results indicate that Hfq is a key regulator involved in *Y. pestis* stress resistance, intracellular survival and pathogenesis. It appears that Hfq acts by controlling the expression of many virulence- and stress-associated genes, probably in conjunction with small noncoding RNAs.

## Introduction

Hfq is proposed to be an RNA-binding protein and was first identified as an *Escherichia coli* protein required for the replication of the RNA phage Qβ [1]. Subsequently, it has been characterized as a global post-transcriptional regulator that acts in numerous bacterial pathways and mediates interactions between many regulatory small RNAs (sRNAs) and their mRNA targets [2], [3]. In most cases, these Hfq-mediated interactions influence the translation or the stability of the target mRNAs. Homologues of *E. coli hfq* have been described in many bacteria [4]. It has also been demonstrated that Hfq contributes to virulence in dozens of pathogenic bacteria [5]–[12].

*Yersinia pestis*, the causative agent of plague, is considered a facultative intracellular pathogen during its early stage of infection. Plague, a deadly disease, is transmitted primarily between fleas and mammals and is spread to humans through the bite of an infected flea or contact with afflicted animals [13]. Analysis of available genome sequences has revealed that *Y. pestis* appears to produce a homologue of Hfq with 88% similarity to *E. coli* Hfq. In this regard, we were interested in understanding the role of Hfq in *Y. pestis* pathogenesis. Using *in vitro* assays, we first demonstrated that Hfq affects a number of phenotypes, including sensitivity to heat, oxidative stress and tolerance to long-term nutrient-limiting and polymyxin B treatment. Then, we examined the role of Hfq in *Y. pestis* virulence using macrophage and mouse infection models. Meanwhile, many Hfq-dependent genes were determined by microarray-based transcriptome analysis, which supported the results concerning the attenuated phenotype of the *hfq* deletion mutant.

## Results

### Phenotypic comparisons between *Y. pestis* WT and Δ*hfq*::Km^R^ mutant

**Figure 1:**
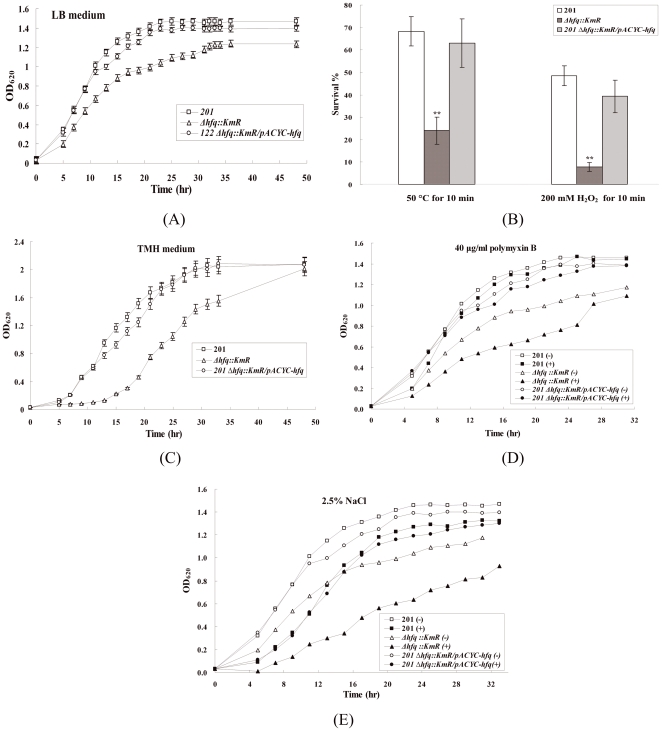
*In vitro* stress resistance of *Y. pestis* WT, Δ*hfq*::Km^R^ and 201 *Δhfq*::Km^R^/pACYC-*hfq* strains. Resistance to heat- or H_2_O_2_-mediated lethality (B) and growth curves of *Y. pestis* WT and Δ*hfq*::Km^R^ strain in TMH medium (C) or in LB media (A) in the presence (+) or absence (−) of 40 µg/ml polymyxin B (D) and 2.5% NaCl (E) at 26°C. **P<0.01, the survival percentage of the Δ*hfq*::Km^R^ mutant was significantly lower than that of *Y. pestis* WT strain 201.

The growth of wild-type (WT) *Y. pestis*, the *hfq* deletion mutant (Δ*hfq*::Km^R^) and its complementary strain (201 *Δhfq*::Km^R^/pACYC-*hfq*) was compared in LB liquid medium at 26°C (Figure 1A). The Δ*hfq*::Km^R^ mutant showed a lower optical density during the entire tested period when compared with the WT strain 201, while 201 *Δhfq*::Km^R^/pACYC-*hfq* showed a growth rate similar to the WT strain. Meanwhile, cell viability of the Δ*hfq*::Km^R^ strain was uncompromised compared to that of the WT strain 201 at the same OD_620_ of 0.7 (data not shown).

Within phagocytes during the early stage of infection, *Y. pestis* must survive stresses such as oxidative agents, high osmolarity, limited nutrition and antibacterial peptides [14]. In addition, the heat shock response might be also elicited from several stresses during growth of the facultative *Y. pestis* within phagocytes. Because of the pleiotropic effects of a deletion of *hfq* in some bacteria [12], the susceptibility to the various stress conditions mentioned above was compared between WT *Y. pestis* and Δ*hfq*::Km^R^ cells. Deletion of the *hfq* gene in *Y. pestis* caused the survival percentage to decrease by about 40% upon exposure to either heat or oxidative stress (Figure 1B). TMH, a chemically defined medium, is used as a nutrition-limiting medium, which provides the essential nutrients but never the rich nutrients that *Y. pestis* requires for *in vitro* growth [15]. The *Y. pestis* Δ*hfq*::Km^R^ mutant showed a longer lag phase (15 hr) after inoculation into fresh TMH medium and reached the stationary phase at the same optical density when compared with the WT strain 201 (Figure 1C); this growth pattern is different from the growth behavior observed in LB medium (rich medium). We also analyzed the survival of the WT and Δ*hfq*::Km^R^ strains during long-term incubation in LB medium containing 40 µg/ml polymyxin B (antibacterial peptide) or 2.5% NaCl (high osmolarity). Growth of the Δ*hfq*::Km^R^ strain was obviously repressed in the presence of polymyxin B compared to that of the WT strain 201 (Figure 1D). Growth retardation of both the WT and Δ*hfq*::Km^R^ strains were observed upon exposure to high concentration of NaCl, but the growth rate difference between the strains was not statistically significant (Figure 1E).

In summary, these data indicated that Hfq contributes to the resistance to heat, oxidative stress, nutrition limitation and antibacterial peptide, but is not required for the resistance of *Y. pestis* to high osmolarity.

### The reduced phagocytosis resistance and intracellular survival of *Y. pestis* Δ*hfq*::Km^R^ mutant

**Figure 2:**
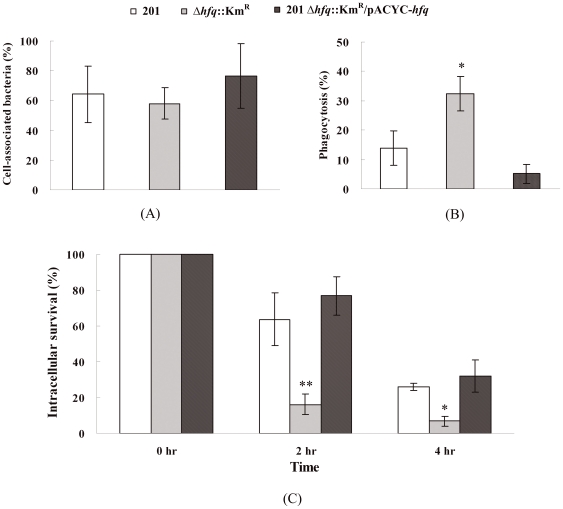
Infection of J774A.1 mouse macrophages with *Y. pestis* WT, Δ*hfq*::Km^R^ and 201 *Δhfq*::Km^R^/pACYC-*hfq* strains. The percentage of cell-associated bacteria (A) was calculated as the number of macrophage cell-associated bacteria divided by the inoculum times 100, while the percent phagocytosis (B) was calculated as the number of intracellular bacteria at the zero point divided by the macrophage cell-associated bacteria times 100. The percent intracellular survival (C) was calculated as the number of intracellular bacteria at the 2^nd^ or 4^th^ hr post-infection divided by that at the zero point. **P<0.01, *P<0.05, the percentage of the Δ*hfq*::Km^R^ mutant was significantly lower than that of *Y. pestis* WT strain 201.

The ability of *Y. pestis* to proliferate in macrophages is likely to be important in the early stages of plague pathogenesis. The increased susceptibility of *Y. pestis Δhfq:*:Km^R^ to several environmental stresses mimicking phagosome microenvironments suggested that Hfq likely played a role in *Y. pestis* antiphagocytosis. To test whether Hfq had any influence on adherence to and survival within phagocytes, J774A.1 murine macrophage cells were infected with *Y. pestis* strain 201, *Δhfq:*:Km^R^ and *Δhfq:*:Km^R^/pACYC-*hfq*. There was no significant difference in the ability of three strains to bind to or associate with J774A.1 macrophages (approximately 60% of the inoculated bacteria) (Figure 2A). In contrast, *Y. pestis Δhfq:*:Km^R^ showed two-fold higher levels of phagocytosis by macrophages than did the WT strain (Figure 2B). Despite starting with relatively high numbers of internalized bacteria, *Y. pestis Δhfq*::Km^R^ cells exhibited substantial drops in viable counts inside J774A.1 (83.9% killed), indicating extensive killing by the macrophages by the 2^nd^ hr post-infection. 63.7% and 76.8% of viable WT and complemented strain, respectively, were recovered over the same period. At the 4^th^ hr post-infection, the survival percentage of *Δhfq*::Km^R^ was statistically lower than both WT and *Δhfq*::Km^R^/pACYC-*hfq* strains (Figure 2C).

These results suggested that Hfq is involved in the phagocytosis and intracellular survival of *Y. pestis* but has no significant impact on adherence to cultivated macrophages.

### The impaired virulence of *Y. pestis* Δ*hfq*::Km^R^ mutant

**Figure 3:**
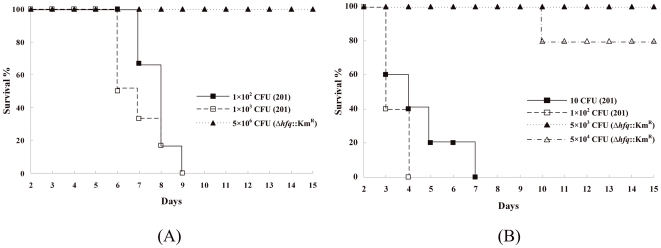
Survival of mice infected subcutaneously (A) or intravenously (B) with *Y. pestis* WT (□ or ▪) and Δ*hfq*::Km^R^ (Δ) strains.

The deletion of the *hfq* gene affected the virulence of *Y. pestis* strain 201. The LD_50_ of both the WT strain 201 and the 201 *Δhfq*::Km^R^/pACYC-*hfq* strain were <10 CFU subcutaneously (*s.c.*), but up to about 5×10^6^ cells of the Δ*hfq*::Km^R^ strain was not lethal to mice inoculated *s.c.* (Figure 3A). The observation suggested that the significantly decreased virulence of the Δ*hfq*::Km^R^ strain is due to the lack of Hfq protein rather than to polar effects caused by the insertion of a kanamycin resistance cassette. For the intravenous route of infection, the LD_50_ of the WT strain 201 was <10 CFU, while only one of five mice was dead on day 10 post-infection with 5×10^4^ cells of Δ*hfq*::Km^R^ (Figure 3B).

**Figure 4:**
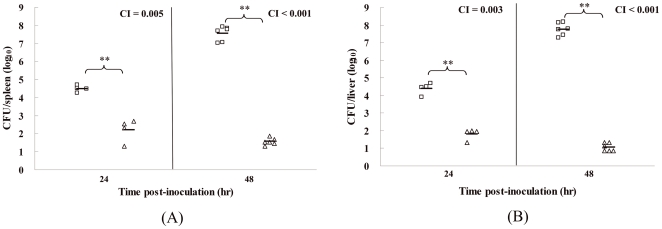
Growth within mice organs after intravenous infection with *Y. pestis* WT (□) and Δ*hfq*::Km^R^ (Δ) strains. **P<0.01, values of *Y. pestis* strain 201 were significantly higher than that of the Δ*hfq*::Km^R^ mutant. The average of log_10_ CFU in the spleen (A) or liver (B) for four or six mice is shown as a horizontal bar; Geometric means of CI values from the respective groups are shown in the graph.

Competitive index (CI) analysis is considered to be a sensitive measure of the virulence attenuation of bacterial pathogens [16], [17]. Competitive assays were performed in mice by intravenously inoculating them with bacteria to examine the *in vivo* fitness of the Δ*hfq*::Km^R^ mutant compared to WT strain 201. We noted that few bacteria of the Δ*hfq*::Km^R^ mutant strain were recovered from the spleen or liver after inoculation with 1×10^4^ CFU, but all the infected animals had a burden of the WT strain approaching 10^7^ to 10^8^ CFU in the spleen or liver at 48 hours post-inoculation (Figure 4). The mean CI values in the spleens were 0.005 at 24 hours and <0.001 at 48 hours (Figure 4A), and similar CI values were obtained in the livers (0.003 at 24 hours and <0.001 at 48 hours) (Figure 4B). The competition experiments demonstrated that the Δ*hfq::*Km^R^ mutant was significantly attenuated when compared to the WT *Y. pestis*. Clearly, the Δ*hfq*::Km^R^ mutant was almost completely overtaken by the population of WT *Y. pestis*, indicating that this mutant was much less competitive *in vivo* than its parental strain.

To determine whether the decline in CI value was due to a generalized growth disadvantage or an inability to compete under nutrient-limiting conditions, *in vitro* competitive assays were carried out in LB or TMH medium. An equal mixture of WT *Y. pestis* and the Δ*hfq*::Km^R^ mutant was diluted 20-fold into either fresh LB or TMH medium and allowed to grow at 37°C for 8 hr. The *in vitro* CI value was then calculated. The results showed that the growth of Δ*hfq*::Km^R^ approximated to that of strain 201 *in vitro* (CI = 0.61 for LB medium or 0.84 for TMH medium). These observations indicated that Hfq could likely be required for *Y. pestis* survival and proliferation in mice.

### Many virulence- or stress-related transcripts affected by Hfq

**Figure 5:**
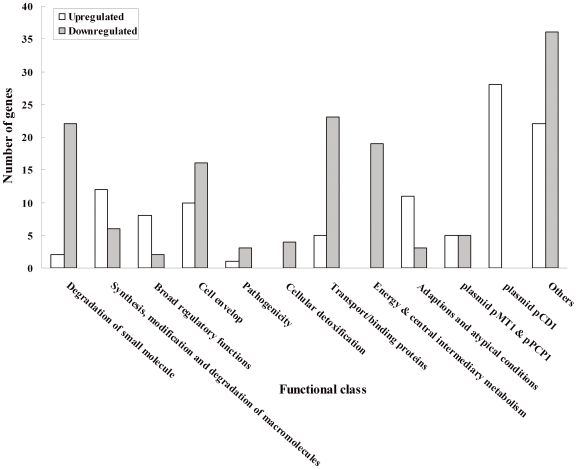
Functional classification of Hfq-dependent genes according to the *Y. pestis* CO92 Genome Project.

To observe the effects of *hfq* deletion on gene expression, DNA microarrays that cover 4005 protein-coding genes of the *Y. pestis* genome [18] were hybridized with total RNA from two independent preparations of the WT and mutant cells. RNA was extracted from *Y. pestis* cells cultivated at 37°C, as the transcription of *hfq* is induced at 37°C and this has proven to produce numerous virulence factors [15], [18]–[20]. In total, 243 genes showed a two-fold or greater difference in transcript abundance in the Δ*hfq::*Km^R^ mutant when compared to the WT strain 201 (Table S1). Among them, 139 genes were down-regulated and the other 104 up-regulated. Functional classification according to the genome annotation of *Y. pestis* CO92 (http://www.sanger.ac.uk/Projects/Y_pestis/) showed that these altered genes belong to more than 13 functional categories (Figure 5). There are four over-represented functional classes consisting of 64 genes, which account for 26.3% of all regulated genes. Approximately 15.8% and 13.7% of the down-regulated genes belong to the classes of degradation of small molecules or energy metabolism, respectively, but only 2.7% and 3.0% (107 and 122 genes) of the whole *Y. pestis* genome belong to these two functional classes, respectively. Similarly, approximately 11.5% and 10.6% of the up-regulated genes belong to the classes of macromolecules metabolism or adaptations and atypical conditions, respectively, but only 4.7% and 0.9% (187 and 36 genes) of the whole *Y. pestis* genome belong to these two functional classes, respectively.

About 23% of all the Hfq-dependent transcripts (56 genes) are hypothesized to be related to pathogenicity or stress adaptation processes, some of which are listed in Table 1. Both *pla*, which encodes the plasminogen activator, and *caf1*, which is responsible for capsule synthesis, were down-regulated in the Δ*hfq*::Km^R^ mutant. KatA is the main scavenger of exogenous H_2_O_2_ in *Y. pestis*, as shown in our previous study [21]. Some genes involved in oxidative stress, including *dps*, *katA*, *katY*, *sodA* and *sodC*, were down-regulated in Δ*hfq*::Km^R^, which is further correlated with the observation that the Δ*hfq*::Km^R^ mutant was obviously impaired in its resistance to high H_2_O_2_ concentration (Figure 1B). Besides that, the *uspA* and *uspB* genes, which are responsible for universal stress, were also down-regulated more than 2-fold. Thus, a possible role in stress responses of *Y. pestis* could be suggested for these gene products, which are involved in the survival of growth-arrested cultures in *E. coli* [22].

**Table 1:** Hfq-dependent genes involved in stress resistance and virulence as determined by microarray and quantitative RT-PCR analysis.

Gene name	Function	Fold change#	
		Microarray	Quantitative PCR
Known virulence factors			
*pla*	coagulase/fibrinolysin precursor	−2.4	−3.3
*caf1R*	putative F1 operon positive regulatory protein	−15.6	−6.8
*caf1M*	putative F1 chaperone protein	−14.7	ND
*caf1A*	putative F1 capsule anchoring protein	−8.3	ND
*caf1*	putative F1 capsule antigen	−9.6	−4.1
*psaE*	Psa type pili regulatory protein	4.1	1.6
*psaA*	pH 6 antigen precursor (antigen 4)	5.3	2.0
*psaB*	chaperone protein PsaB precursor	5.8	ND
*rovA*	MarR-family transcriptional regulatory protein	6.5	2.0
*hmsT*	HmsT protein	2.4	2.0
Detoxification of oxidative agents			
*katA*	catalase	−4.3	−3.7
*dps*	putative DNA-binding protein	−3.1	−3.0
*katY*	catalase-peroxidase	−3.3	−2.0
*sodC*	superoxide dismutase [Cu-Zn] precursor	−4.5	−1.9
*sodA*	superoxide dismutase [Mn]	−3.2	−1.5
Heat shock proteins			
*hslU*	ATP-binding heat shock protein	2.8	2.1
*hslV*	heat shock protein	2.7	ND
*hslO*	heat-shock chaperonin	2.3	1.5
*hslR*	heat shock protein 15	2.4	ND
*htpX*	putative heat shock protein	2.2	–
*lon*	ATP-dependent protease La	2.1	–
*htpG*	heat shock protein	3.0	1.4
Universal stress			
*uspB*	universal stress protein B	−2.0	−1.4
*uspA*	universal stress protein A	−2.5	−1.5
pMT1-unknown			
YPMT1.34	hypothetical protein	118.4	38.9
YPMT1.34A	hypothetical protein	75.4	37.0

# Fold change represents the mRNA abundance in *Y. pestis* Δ*hfq*::Km^R^ mutant compared with that in the WT strain. Positive numbers represent increases, while negative numbers represent decreases.

“ND”, not detected since they belong to the same operon with the adjacent genes.

“–”, undetectable due to low abundance of mRNA or low efficiency of the corresponding primer pairs.

Of the data available for 57 genes on plasmid pCD1, almost 50% (28 genes) displayed statistically significant increases at the mRNA level in the Δ*hfq*::Km^R^ mutant. Since *sopAB*, which encodes putative plasmid partitioning control proteins, was also up-regulated, it cannot be excluded that Hfq has an impact on the replication process of this plasmid. Surprisingly, several genes involved in the heat shock response such as *hslUV*, *hslOR*, *htpG*, *htpX* and *lon* were up-regulated in the Δ*hfq*::Km^R^ mutant. In addition, *psaEF* and *psaABC*, which are responsible for the synthesis of the pH 6 antigen [23], [24], and *rovA*, which plays a role in virulence regulation [25], were also found to be up-regulated upon deletion of the *hfq* gene from *Y. pestis*. The drastic up-regulation of YPMT1.34 and YPMT1.34A (more than 100-fold) is difficult to explain because no known functions have been attributed to these genes.

All the operons or genes listed in Table 1 were chosen to compare data between microarray and quantitative RT-PCR techniques. As previous studies indicated, the relative values of each gene measured by RT-PCR were always lower than those measured by microarray. For example, five differentially regulated genes including *psaE*, *sodA*, *hslO*, *uspB* and *uspA* were found to only be regulated 1.4- to 1.6-fold by RT-PCR. However, a high level of concordance (r = 0.97) was observed between the microarray results and the real-time RT-PCR data (Figure 16), which confirms the reliability of the data produced in this study.

**Figure 6:**
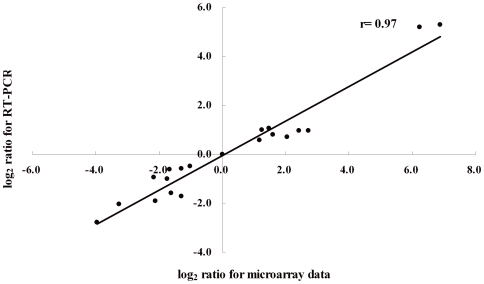
Comparison of transcription measurements by microarray and real-time PCR assays.

## Discussion

The RNA-binding protein Hfq was recently assumed to be a key regulator that plays an important role in pleiotropic effects, including stress resistance and virulence, in both Gram-negative bacteria [5]–[7], [9], [10], [12], [26] and Gram-positive bacteria [11]. Therefore, we speculated that Hfq is required for *Y. pestis* survival and proliferation in mice. In this study, we have shown that deletion of the *hfq* gene resulted in a significant reduction in *Y. pestis* virulence as evidenced by an increased LD_50_. The competitive assays indicated that, in the absence of Hfq, *Y. pestis* cells might be quickly eliminated by the host immune system.

It is believed that *Y. pestis* survives and replicates within macrophages during the early stages of infection at peripheral host sites [14], [27]. The moiety that escapes from macrophages can multiply outside of host cells and eventually cause systemic infection. The phagosomal microenvironments, which can be subject to heat shock, oxidative agents, nutrition limitation, high osmolarity and killing or inhibiting activities of antibacterial peptides *etc*., are harmful to the survival and proliferation of *Y. pestis in vivo*. Based on this notion, the importance of *Y. pestis* Hfq under stress conditions was examined by comparing the growth or survival characteristics of the Δ*hfq*::Km^R^ mutant to those of the wild-type strain. We found that the Δ*hfq*::Km^R^ mutant is more sensitive to exogenous H_2_O_2_, heat, nutrient limitation and the antibacterial cationic peptide polymyxin B, which *Y. pestis* might encounter during its infection. It is demonstrated that Hfq seems to be involved in stress resistance, which determines *Y. pestis* persistence *in vivo.* These results suggest that Hfq is likely required for *Y. pestis* survival within phagocytes and thus influences the ability of *Y. pestis* to elicit a systemic infection.

Our data indicated that Hfq might be involved in *Y. pestis* persistence within macrophages, especially during the initial stage of infection, which is consistent with the hypothesis inferred from environmental stresses *in vitro*. Even though the exact impact of Hfq on the intracellular growth of *Y. pestis* remains to be characterized, the high susceptibility of *Δhfq*::Km^R^ to several stressful conditions *in vitro* might partially explain the poor survival of the mutant within J774A.1 macrophages at 2^nd^ hr and 4^th^ hr post-infection.

In order to survive and grow within the hostile environments of host organisms, *Y. pestis* must acclimatize to environmental changes and respond quickly by adjusting the expression of stress- and virulence-associated genes. In this study, the potential connections between Hfq, stress resistance and the pathogenesis of *Y. pestis* were further supported by the observation that the deletion of *hfq* caused significant changes in the gene expression profile of strain 201. In some bacteria, Hfq has proved to influence the expression of virulence genes by altering the stability of their mRNAs [26], [28]–[31]. We therefore anticipated different expression of the genes encoding virulence factors in the Δ*hfq*::Km^R^ strain when compared to the WT strain 201 by microarray-based transcriptome analysis. Interestingly, Hfq was shown to cause significant alterations in the expression of some important virulence factors such as Pla, F1 capsular antigen and pH 6 antigen etc. *Y. pestis* is a species evolved from *Y. pseudotuberculosis*, and it has acquired an exceptional pathogenicity potential [32]. The two *Y. pestis*-specific plasmids are pMT1 and pPCP1, which were acquired during speciation of *Y. pestis* from *Y. pseudotuberculosis*. In addition to its role in flea-borne transmission, Pla on plasmid pPCP1 has been shown to contribute to the pathogenesis of bubonic and primary pneumonia plague [33]–[36]. The capsular antigen F1 on plasmid pMT1 and the pH 6 antigen promote resistance to phagocytosis and help the bacteria escape host defense mechanisms [37], [38]. Their differential expression might partially account for the reduced phagocytosis resistance of the *Y. pestis hfq* deletion strain that was shown in our study.

Modulation of the virulence-associated loci might partially account for the attenuated virulence of the Δ*hfq*::Km^R^ mutant. Besides that, the Hfq-dependent genes involved in stress resistance were likely to be regulated in order to facilitate the resistance and adaptation of *Y. pestis* to hostile host environments. Heat shock proteins in *Y. pestis* express at 45°C, as described previously [39], while the present observation showed that in the absence of *hfq* their activation occurred even at 37°C due to increased sensitivity to heat stimuli. Hfq also affected stress-inducible genes involved in the detoxification of oxidative agents and the universal stress response, which might contribute to the diminished capacity of the *Y. pestis* Δ*hfq*::Km^R^ mutant to tolerate different environmental stresses and to survive within cultured J774A.1 macrophages.

Hfq is also important for protein translation by changing ribosome accessibility through mRNA-sRNA pairing [40]–[43]. Further attempts are being made in our lab to determine Hfq-dependent changes in *Y. pestis* protein expression. The modulation of bacterial pathogenicity by Hfq has been shown to be associated with small non-coding regulatory RNAs [26], [44]. There is little information regarding sRNAs in *Y. pestis* except for *gcvB*, which was recently reported by Mcarthur *et al* [45]. Thus, further studies will be necessary to determine if sRNAs are associated with the *hfq* defective virulence phenotype in *Y. pestis*.

## Materials and Methods

### Bacterial strains and growth media

The *Y. pestis* WT strain 201 and the Δ*hfq::*Km^R^ mutant were used in this study. Strain 201 was isolated from *Microtus brandti* in Inner Mongolia, China. Its major phenotypes are F1^+^ (able to produce fraction 1 capsule), LcrV^+^ (presence of V antigen), Pst^+^ (able to produce pesticin) and Pgm^+^ (pigmentation on Congo-red media). The genome contents of strain 201 were identical to *Y. pestis* strain 91001 according to our previous DNA microarray-based comparative genomic analysis. Both strain 201 and strain 91001 belong to a newly established *Y. pestis* biovar, microtus [46], which is supposed to be avirulent in humans but highly lethal in mice [47].

The *hfq* deletion mutant of *Y. pestis*, Δ*hfq::*Km^R^, was constructed by replacing the entire 306-bp *hfq* gene with the *kan* cassette by means of Red homologous recombination. The WT strain 201 carrying plasmid pKD46, which could express the highly efficient Red homologous recombination system, was used as the competent host. The PCR fragment carrying the *kan* cassette flanked by regions homologous to the *hfq* gene was electroporated into the competent cells. The recombinant colonies were selected due to their kanamycin resistance. To obtain a strain in which *Δhfq*::Km^R^ is complemented, plasmid pACYC184, which contains a PCR fragment covering a region from 300 bp fragment upstream to 200 bp downstream of the *hfq* gene, was introduced into *Y. pestis* Δ*hfq*::Km^R^.

### Stress resistance assays

*Y. pestis* WT strain 201 and the Δ*hfq::*Km^R^ mutant were grown in LB medium at 26°C to middle exponential phase (OD_620_ = 0.7∼0.9). The bacterial cultures were harvested, resuspended in 0.85% NaCl and adjusted to an OD_620_ of 0.7. Cells were diluted 1:20 in the chemically defined medium TMH (nutrition limitation) [15] or LB medium containing 2.5% NaCl or 40 µg/ml polymyxin B. Cells were then incubated at 26°C. Bacterial growth was monitored by measuring absorbance at OD_620_. For heat treatment, cells at mid-log phase were transferred to 50°C for 10 min. For oxidative resistance determination, mid-log cells grown at 26°C were transferred to 37°C for 3 hr and then treated with 200 mM H_2_O_2_ for 10 min. Dilutions of the cell suspension were plated on Hottinger agar to determine the number of viable bacteria. All the experiments were performed in three independent cultures. For heat, H_2_O_2_ and nutrient-limiting tolerance experiments, results are expressed as the mean percentage±standard deviation from three independent experiments. The growth curve for polymyxin and NaCl treatment consists of the means of results from three independent experiments with similar results.

### Macrophage infection

Since *Y. pestis* can replicate in J774A.1 macrophages [48], exponential growth of *Y. pestis* strain cells at 26°C followed by a 3 hr incubation at 37°C were used to infect these macrophages at a multiplicity of infection (MOI) of ∼50. After addition of bacteria, the tissue culture plates were centrifuged at 500 *g* for 5 min to facilitate bacterial contact with macrophage cells. The macrophages were washed twice with PBS after a 30 min incubation and the number of total macrophage cell-associated bacteria was determined. Then fresh medium containing 50 µg of gentamicin per ml (Ameresco, Solon, OH, USA) was added for 30 min to each well to kill extracellular bacteria and the number of intracellular bacteria was determined as a control (referred to as the zero point). The cells were washed twice with PBS, and fresh medium containing 10 µg of gentamicin per ml was added to the cells. The cells were incubated at 37°C with gentamicin treatment and at the zero point, hour 2, and hour 4, the macrophages were washed and lysed, the cell lysates were collected and viable bacteria was counted. Duplicate samples were taken at all time points and each experiment was repeated twice on different days. The results are represented as the averages of data from two experiments.

### Mouse infections

Groups of five or six 6-week-old female BALB/c mice were injected subcutaneously or intravenously with 5 to 5×10^6^ bacteria. Mortality was recorded daily for 14 days. LD_50_ values were calculated by the Reed-Muench equation [49].

*Y. pestis* WT strain 201 and the Δ*hfq*::Km^R^ mutant were grown in LB medium at 26°C overnight. The bacterial cultures were washed and diluted to 2×10^5^ cells per ml in 0.85% NaCl, and 0.1 ml of a 1∶1 mixture of the two bacterial strains was used to infect five BALB/c mice intravenously. For CI determination, the infected mice were sacrificed after 24 or 48 hrs and bacterial cells were recovered from livers and spleens. Serial dilutions were plated on Hottinger agar containing or not containing kanamycin to determine the colony-forming units (CFUs) per organ. The CI value was calculated as the ratio of the number of mutant/WT bacteria recovered [50]. The data were analyzed by Student's *t*-test, with *P*<0.05 considered statistically significant. All mouse experiments were carried out according to the Guidelines for the Welfare and Ethics of Laboratory Animals of Beijing.

### Microarray-based transcriptome and quantitative RT-PCR analysis

*Y. pestis* WT strain 201 and the Δ*hfq*::Km^R^ mutant were grown at 26°C in LB medium to middle exponential phase and then transferred to 37°C for 3 hr. Two independent bacterial cultures of strain 201 (control condition) and the Δ*hfq*::Km^R^ mutant (test condition) were considered biological replicates for RNA isolation. Four separate labeled probes were made for each RNA preparation as technical replicates. cDNA synthesis and Cy3 or Cy5 dye labeling were performed as described previously. Pairwise comparisons were made using dye swaps to avoid labeling bias. Microarray-based hybridization was performed as previously described [39]. For data filtering and data analysis, spots with background-corrected signal intensity (median) in both channels that were less than two fold of background intensity (median) were rejected from further analysis. Data normalization was performed on the remaining spots by total intensity normalization methods. The normalized log_2_ ratio of test/reference signal for each spot was recorded. Genes with less than three data points were considered unreliable, and their data points were discarded as well. The averaged log_2_ ratio for each remaining gene on the eight replicate slides was ultimately calculated. Significant changes in gene transcription level were identified with SAM software [51] using one class mode. The array data was deposited in Gene Expression Omnibus in accordance with MIAME guidelines (GEO accession number GSE15579).

For quantitative RT-PCR, cDNA was generated using 5 µg of total RNA and 3 µg of random hexamer primers with the Superscript II system. Gene-specific primers were designed using ArrayDesigner 2.0 software (Table S2). All primer pairs produced a 150∼200 bp amplicon when *Y. pestis* genomic DNA was used as the template for PCR. Real-time PCR was performed in duplicate for each RNA preparation using the LightCycler system (Roche) with an appropriate dilution of cDNA as a template. On the basis of the standard curve of 16S rRNA, the relative mRNA level was determined by calculating the threshold cycle (ΔCt) of each gene by the classic ΔCt method. Quantification of 16S rRNA was also used to normalize the values all the other genes in the RT-PCR experiment.

## Supporting Information

Table S1 Hfq-dependent genes of *Y. pestis* as determined by microarray analysis.
(0.07 MB XLS)

Table S2 Oligonucleiotide primers used for real-time quantitative RT-PCR.
(0.05 MB DOC)
