# Supplementary material for: Involvement of the Post-Transcriptional Regulator Hfq in Yersinia pestis Virulence
Source: PLoS One. 2009 Jul 10;4(7):e6213. doi: 10.1371/journal.pone.0006213 (PMC2704395; doi:10.1371/journal.pone.0006213)
Supplement: Table S2 — Oligonucleiotide primers used for real-time quantitative RT-PCR. (0.05 MB DOC) [file pone.0006213.s002.doc]

**Table S2 Oligonucleiotide primers used for real-time quantitative RT-PCR.**

| **Gene ID** | **Gene Name** | **Sense primer (5'-3')** | **Antisense primer (5'-3')** |
| --- | --- | --- | --- |
| YPMT1.81c | *caf1R* | AATAGGCATTCCGACATATTCC | TTCATGCTAAAACAGATGACTG |
| YPMT1.84 | *caf1* | AGATGCCGCGGGTGATC | TCACTTTTGTAGTGAATTGGTGGTTAT |
| YPPCP1.07 | *pla* | ATGGATGACTACGACTGGATG | TATGAACCACCTGTAGCTGTC |
| YPO1301 | *psaE* | GTCGTCTCCCCAACATCAC | TTCGGTGCTGCCATCATC |
| YPO1303 | *psaA* | CAAGCAGGGAAACACATTC | ACCAACATAGTCACCATCG |
| YPO2374 | *rovA* | TTACCACCAGAGCAATCACAG | ATCACGCCATCAACCTGTTC |
| YPO0425 | *hmsT* | GTTCCCTGTGGTGATGATTGC | TGCCCAAAGTAGACCGATGAG |
| YPO1207 | *katA* | TGTCCGCTTCTCCACTGTG | GCAGGTTGGTACGAGGGTC |
| YPO3319 | *katY* | GTGTTGACGAATACGCCAGG | ACTTCCGCAACCGCTCTG |
| YPO2510 | *dps* | CTCGGTGGTGTGGCATTG | CGGGAAGCGGCAGTAAAC |
| YPO3375 | *sodC* | ATGGCTGGCATGAATGATAAGG | AGATGTGGCGTAAACAGTAAGC |
| YPO0139 | *hslO* | ATAAAGCGGCGGCAGGTG | GGAGCAGGTACAGCGGAAG |
| YPO0105 | *hslU* | TGAAATTGCCCGCCGTCTG | CAACTCTTCCGCCCGATAGC |
| YPO1706 | *htpX* | GATTATGGCGGGTCTGTTTGG | ACTTGTGGCATGGCGATCC |
| YPO3119 | *htpG* | CTGGCAACAATTCGGCATGG | TACTCTTCGCAGCAGCATAGC |
| YPO3155 | *lon* | AGAAAGAACTGGGCGAGATGG | TGCTGCGGCTATTCCAAGG |
| YPO3970 | *uspA* | TGACGTTAATCTCGGCGATATG | ATCCTGATGATGACCACACAAG |
| YPO3969 | *uspB* | CTATTCATCTCTGCGTGCTCTG | CTCACAGCGGCGGATAAATTC |
| YPMT1.34 | – | GCAGCCAACAATGCGACTC | AACGCTTCAGGTGCTTGTTTG |
| YPMT1.34a | – | TCGCTGGCGTTAACATCC | ACATCGGATTCTTTGATTCCTG |
| 16s RNA | 16s RNA | TTACCTACTCTTGACATCCAC | GCTGGCAACAAAGGATAAG |
